# An Extended DIY Light-sheet Microscope Enabling Four-color Volumetric Imaging of Whole Biological Samples

**Published:** 2026-08-04

**Authors:** YUKI NOZAWA, STEVEN EDWARDS, YURI SAITO, HJALMAR BRISMAR, KOHEI OTOMO, ETSUO A. SUSAKI

**Affiliations:** 1 Department of Biochemistry II, Faculty of Medicine, Juntendo University, Tokyo, Japan; 1 Department of Biochemistry II, Faculty of Medicine, Juntendo University, Tokyo, Japan; 2 Nakatani Biomedical Spatialomics Hub, Graduate School of Medicine, Juntendo University, Tokyo, Japan; 2 Nakatani Biomedical Spatialomics Hub, Graduate School of Medicine, Juntendo University, Tokyo, Japan; 3 Science for Life Laboratory, KTH Royal Institute of Technology, Solna, Sweden; 3 Science for Life Laboratory, KTH Royal Institute of Technology, Solna, Sweden; 4 Department of Biochemistry and Systems Biomedicine, Graduate School of Medicine, Juntendo University, Tokyo, Japan; 4 Department of Biochemistry and Systems Biomedicine, Graduate School of Medicine, Juntendo University, Tokyo, Japan; 5 Science for Life Laboratory, Karolinska Institute, Stockholm, Sweden; 5 Science for Life Laboratory, Karolinska Institute, Stockholm, Sweden; 6 Biophotonics Research Division, National Institute for Physiological Sciences (NIPS), National Institutes of Natural Sciences, Aichi, Japan; 6 Biophotonics Research Division, National Institute for Physiological Sciences (NIPS), National Institutes of Natural Sciences, Aichi, Japan; 7 Biophotonics Research Group, Exploratory Research Center on Life and Living Systems (ExCELLS), National Institutes of Natural Sciences, Aichi, Japan; 7 Biophotonics Research Group, Exploratory Research Center on Life and Living Systems (ExCELLS), National Institutes of Natural Sciences, Aichi, Japan; 8 Laboratory of Cell Biology, Biomedical Research Core Facilities, Juntendo University, Tokyo, Japan; 8 Laboratory of Cell Biology, Biomedical Research Core Facilities, Juntendo University, Tokyo, Japan

**Keywords:** tissue clearing, descSPIM, 3D imaging, light-sheet microscopy, multicolor imaging

## Abstract

**Objectives:**

A combination of tissue clearing technology and light-sheet fluorescence microscopy enables volumetric analysis of intact biological specimens. To analyze complex spatial organizations of molecules and cells, the number of imaging channels available for simultaneous visualization from a single sample is a key parameter. This study aimed to expand the previously reported DIY compact light-sheet microscope, descSPIM, for four-color volumetric imaging of whole biological samples.

**Materials:**

A four-color-labeled optical phantom and an entire E12.5 mouse embryo labeled with a nuclear stain and three distinct antibodies were used.

**Methods:**

We modified the previously reported DIY compact light-sheet microscope, descSPIM, for four-color volumetric imaging. Chromatic aberration was evaluated across the visible wavelength range (400-700 nm) using the four-color-labeled optical phantom. Four-color three-dimensional imaging was then performed on the entire E12.5 mouse embryo.

**Results:**

The chromatic aberration of the extended descSPIM was mostly negligible across the visible wavelength range (400-700 nm). The extended system also enabled four-color three-dimensional imaging covering the entire E12.5 mouse embryo.

**Conclusions:**

Together, these results validate the reliability and expandability of the extended system, supporting its broader use for multicolor imaging of cleared tissue samples.

## Introduction

The combination of tissue clearing technology and light-sheet fluorescence microscopy (LSFM) emerged in the 1990s and matured into a method capable of 3D cellular-resolution imaging of whole organs by the late 2000s^1, 2)^. Early LSFM architectures, including selective-plane illumination microscopy (SPIM) and digitally scanned light-sheet microscopy (DSLM)^3, 4)^, were originally developed for live imaging of small model organisms. Following the development of efficient tissue clearing techniques, LSFM has been widely adopted for three- dimensional (3D) imaging of whole cleared biological samples. Although several commercial LSFM systems for cleared sample imaging provides high performance, many remain prohibitively expensive, limiting their accessibility in standard laboratory settings. To improve the situation, several open-source LSFM platforms have been developed. These include early platforms such as OpenSPIM and OpenSpin Microscopy^5, 6)^, followed by systems designed specifically for large cleared specimens, such as mesoSPIM^7, 8)^. While these systems have expanded access to LSFM devices, their construction and operation often require substantial optical expertise. Consequently, they are primarily intended for imaging facilities rather than routine use by biomedical end users. To address this gap, we previously developed descSPIM (desktop equipped SPIM for cleared specimens) as a user-friendly LSFM platform that can be constructed at low cost with minimal technical expertise while enabling cellular-resolution imaging at the whole-organ scale^9)^. This design lowered the barrier to three-dimensional imaging of cleared tissues in standard laboratory environments.

One limitation of the previous descSPIM configuration (descSPIM-basic) was its limited color channel expandability (2-3 colors), a trade-off accepted to maintain simplicity and cost efficiency. As biological research increasingly requires the simultaneous observation of multiple molecular components, expanding multicolor imaging capabilities of the descSPIM platform has become necessary. Specifically, minimizing chromatic aberration is critical when incorporating additional color channels across a broader wavelength range. Therefore, there remains a need for optical strategies that allow for straightforward and accurate multicolor imaging without substantially increasing system complexity or the technical burden on the end user, while maintaining cellular resolution and whole-organ imaging capability.

Here, we present an extended descSPIM system designed for four-color volumetric imaging across the 400 nm to 700 nm wavelength range. To assess the optical performance, we also established a method to evaluate chromatic aberration between channels throughout the visible spectrum. The developed system enabled four-color imaging of an entire E12.5 mouse embryo, visualizing multiple molecular expression patterns within their spatial context.

## Materials and Methods

### descSPIM construction

The extended descSPIM system was constructed based on the descSPIM building guide provided on our GitHub (https://github.com/dbsb-juntendo/descSPIM/tree/main/descSPIM-basic), with minor modifications. The detection optics comprised a 1 × objective lens (Thorlabs, TL1X-SAP) and a 200- mm tube lens (Thorlabs, TTL200-A), yielding an effective magnification comparable to the descSPIM-basic configuration. Illumination optics used a 500-mm cylindrical lens for creating a light-sheet illumination. To expand the number of color channels, a 405 nm laser source was added to 488, 561, and 647 nm excitation wavelengths used in the descSPIM-basic. The fluorescence signal was passed through a bandpass or a longpass filter (Edmund Optics, #84-742, 425 nm-; Thorlabs, FBH520-40, 500-540 nm; Edmund Optics, #84-745, 575 nm-; and Edmund Optics, #84-747, 675 nm-) before the detector.

### PSF measurement

The point spread function (PSF) was evaluated by measuring 3D fluorescence images of φ1 µm Nile Red-labeled FluoSpheres™ Carboxylate-Modified Microspheres (Invitrogen™, Thermo Fisher Scientific, #F8819). The beads were embedded in 2% agarose gel prepared with CUBIC-R+. Excitation wavelength was 561 nm, and fluorescence signals were collected using a 600-nm long-pass filter. From 3D image stacks of a single bead, full-width at half maximum (FWHM) values of lateral and axial fluorescence intensity profiles were estimated as spatial resolutions.

### Chromatic aberration evaluations using a multicolor optical phantom

Chromatic aberration was assessed using a custom multicolor optical phantom prepared from Protein A/G agarose beads (Santa Cruz Biotechnology, #sc-2003). The beads were incubated with a mixture of fluorescently labeled secondary antibodies added at an equal mass ratio of 1:1:1:1 (w/v). The antibody mixture was consisted of goat anti-mouse Alexa Fluor^®^ 405 (Invitrogen™, Thermo Fisher Scientific, #A31553), donkey anti-goat Alexa Fluor^®^ 488 (Invitrogen™, Thermo Fisher Scientific, #A11055), goat anti-rabbit Alexa Fluor^®^ 594 (Invitrogen™, Thermo Fisher Scientific, #A11037), and donkey anti-rabbit Alexa Fluor^®^ 647 (Jackson ImmunoResearch, #711-605-152). After incubation, the beads were thoroughly washed with PBS, embedded in a PBS-based gel, and placed in a four-sided transparent plastic cuvette (Bio-Rad, #1702415) for multichannel imaging. The multichannel imaging was performed using the following excitation wavelength-fluorophore pairs: 405 nm- Alexa Fluor^®^ 405, 488 nm-Alexa Fluor^®^ 488, 561 nm- Alexa Fluor^®^ 594, and 647 nm-Alexa Fluor^®^ 647. For quantitative evaluation of chromatic aberration, the centroid position of each bead was estimated independently for each fluorescence channel using Fiji (ImageJ)^10)^. For each bead, an oval region of interest (ROI) was manually drawn to enclose the fluorescent signal distribution, and the unweighted centroid was calculated independently in the lateral and axial planes using Fiji (ImageJ)^10)^. The 488 nm channel was used as a reference, and centroid displacements (Δ*x*, Δ*y*, and Δ*z*) were measured for the other channels. Lateral chromatic aberration was evaluated using Δ*x* and Δ*y* in the *xy* plane, while axial chromatic aberration was assessed using Δ*z*. Measurements were performed for three independent beads (N = 3). Individual measured values were plotted as data points, and the median value was indicated by a bar for each channel. The measured chromatic displacements were interpreted in relation to the spatial sampling of the imaging system, with a pixel size of 3.45 µm in the *xy* plane and a *z*-step size of 12 µm.

### Mouse embryo preparation

Timed-pregnant ICR mice (Slc:ICR; Japan SLC, Inc.) were purchased from Sankyo Labo Service Corporation (Tokyo, Japan). Pregnant dams were humanely euthanized by CO_2_ inhalation. Embryos at E12.5 were recovered by laparotomy under a stereomicroscope without perfusion. The embryos were immersed in 4% paraformaldehyde in phosphate-buffered saline (PBS) for 24 h, followed by extensive rinsing in PBS. All procedures involving animals were approved by the Institutional Animal Care and Use Committee of Juntendo University (approval nos. 2025248 and 2026291) and performed in accordance with institutional regulations and the Japanese Act on Welfare and Management of Animals.

### Tissue clearing, staining and embedding

Embryonic samples were cleared using a CUBIC-based tissue clearing workflow adapted from previously reported protocols, with minor modifications optimized for embryonic specimens^11, 12)^. Delipidation was achieved by incubation in CUBIC-L at 37 °C overnight, followed by an oxidative bleaching step using 3% H_2_O_2_ in 150 mM NaCl for 2 days to reduce residual pigmentation and background signals associated with blood components. After the treatments, multicolor labeling was conducted following the CUBIC-HistoVIsion 2.0 protocol^13)^. Nuclear labeling was performed with POPO™- 1 Iodide (Thermo Fisher Scientific/Invitrogen, #P3580), and immunolabeling was carried out using TAG1-FITC, αSMA-Alexa Fluor^®^ 568, and RUNX2-Alexa Fluor^®^ 647. These primary antibodies were directly conjugated with these fluorophores by using Lightning-Link^®^ Conjugation Kit (Abcam, #ab188285, #ab269821, and #ab269823) according to the supplier's protocol. The stained specimens were extensively washed, briefly post-fixed in 1% PFA in HEPES-TS (HEPES buffer with Triton X-100 and saline), and subsequently immersed in CUBIC-R+(N) for refractive-index matching. For imaging, cleared embryos were immobilized by embedding in CUBIC-R-agarose^12)^. The embedded samples were mounted in four-sided transparent plastic cuvettes (Bio-Rad, #1702415) and subjected to the LSFM imaging.

### Image acquisition and rendering

All imaging data were acquired as z-stack images and saved in either 12-bit or 8-bit TIFF format. For multichannel imaging, a *z*-stack was acquired sequentially for each channel using Thorlabs Kinesis^®^ and ThorCam™ software. Imaging was performed with a *z*-step size of 15 µm. POPO-1 was excited at 405 nm with a laser power of 100 mW, whereas TAG1-FITC, αSMA-AF568, and RUNX2- AF647 were excited at 488, 561, and 647 nm, respectively, each at a laser power of 10 mW. The exposure time was set to 300 ms for all four channels. Image visualization and rendering were performed using Fiji (ImageJ)^10)^. The lateral pixel size was determined by the CMOS camera specifications and set to 3.45 µm per pixel. The axial step size (*z*-interval) was defined based on the sample stage translation speed and exposure time, and was calculated as the distance traveled by the sample stage per second divided by the exposure duration. This acquisition scheme enabled consistent voxel dimensions across all datasets used for subsequent visualization and analysis.

## Results

### Optical characterization and chromatic aberration assessment of the system

**Figure 1:**
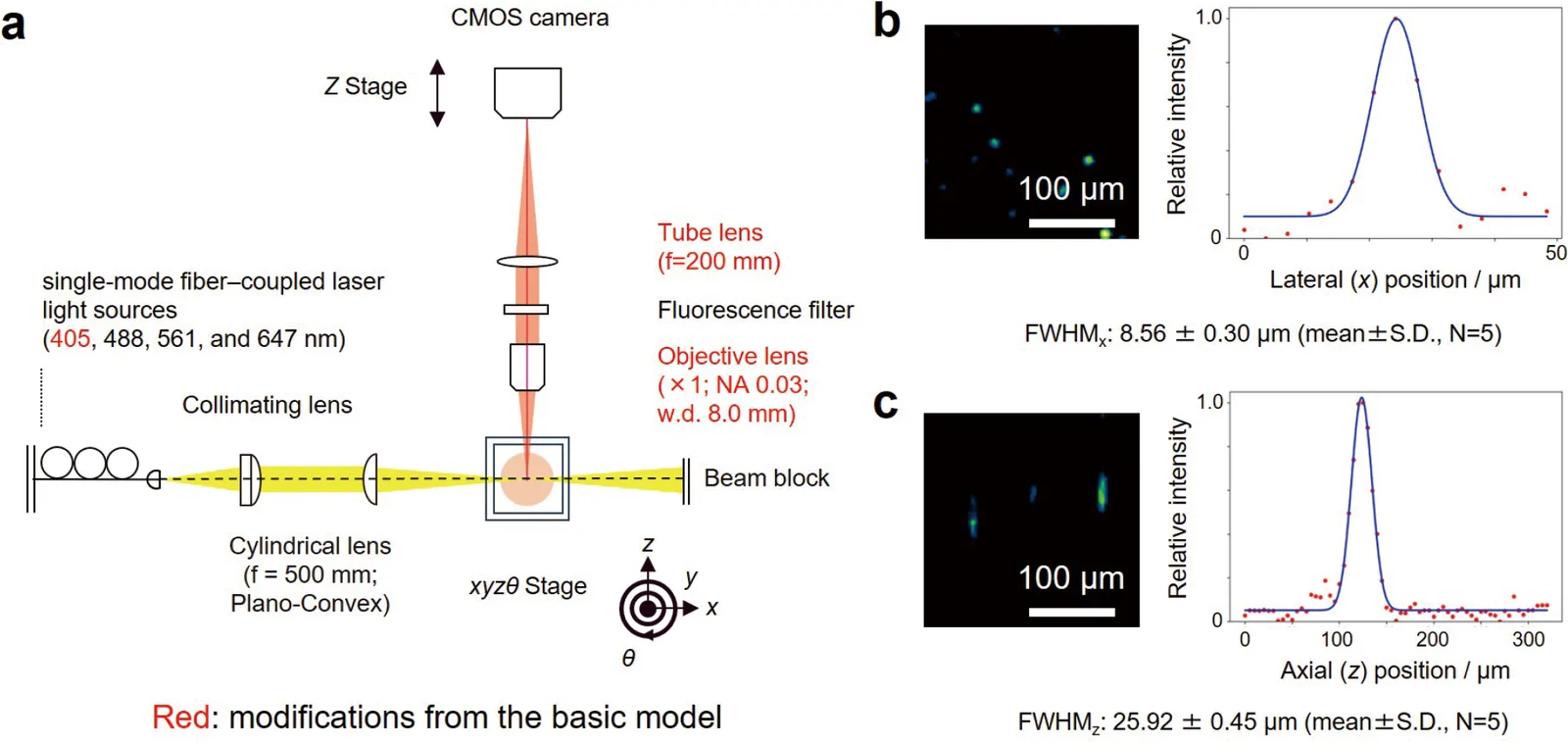
Optical specifications and chromatic aberration assessment of the descSPIM **a.** Optical layout of the alternative descSPIM configuration used in this study. Compared with the previously reported descSPIM-basic model, the detection optics were changed from a 2 × objective lens (NA 0.10, working distance 56 mm) with a 100 mm tube lens to a 1 × objective lens (NA 0.03, working distance 8 mm) with a 200 mm tube lens, while maintaining the same effective magnification (1 ×). The excitation wavelengths were also modified from 488, 515, 561, and 647 nm to 405, 488, 561, and 647 nm. A cylindrical lens (f = 500 mm) was used to generate the excitation light sheet. **b.** Lateral resolution of the descSPIM. **c.** Axial resolution of the descSPIM, which is comparable to that of the basic model (full field of view mode, f = 500 mm cylindrical lens).

The original descSPIM-basic used detection optics composed of a 2 × objective lens (NA: 0.10, working distance: 56 mm) and a tube lens with a focal length of 100 mm, resulting in an effective magnification of 1 ×^9)^. In the extended system, the detection optics were replaced with a 1 × objective lens (NA: 0.03, working distance: 8 mm) and a 200 mm focal length tube lens while maintaining the same effective magnification (Figure 1a). This modification broadens the range of detection optics that can be incorporated into the descSPIM design. On the other hand, we did not change the illumination optics configured using a cylindrical lens with a focal length of 500 mm, corresponding to full field of view mode in the descSPIM-basic^9)^. Further, we incorporated a 405 nm laser source for this extension. While UV-excited fluorophores are known to be susceptible to scattering and typically unsuitable for deep 3D imaging, the use of the bright blue nuclear stain POPO-1 and our refined 3D staining technique made this expansion possible (see below).

To evaluate the optical resolution of the extended descSPIM, we performed point spread function (PSF) measurements (Figure 1b, c). The lateral resolution was lower (FWHM: 8.56 ± 0.30 µm) than that of the descSPIM-basic model (FWHM: 4.20 ± 0.20 µm) due to the lower NA of the detection objective lens (Figure 1b). The axial resolution remained comparable to the basic model under the same full field of view illumination conditions (Figure 1c). Consequently, the extended system maintains cellular resolution across an effective field of view covering the entire CMOS sensor area.

**Figure 2:**
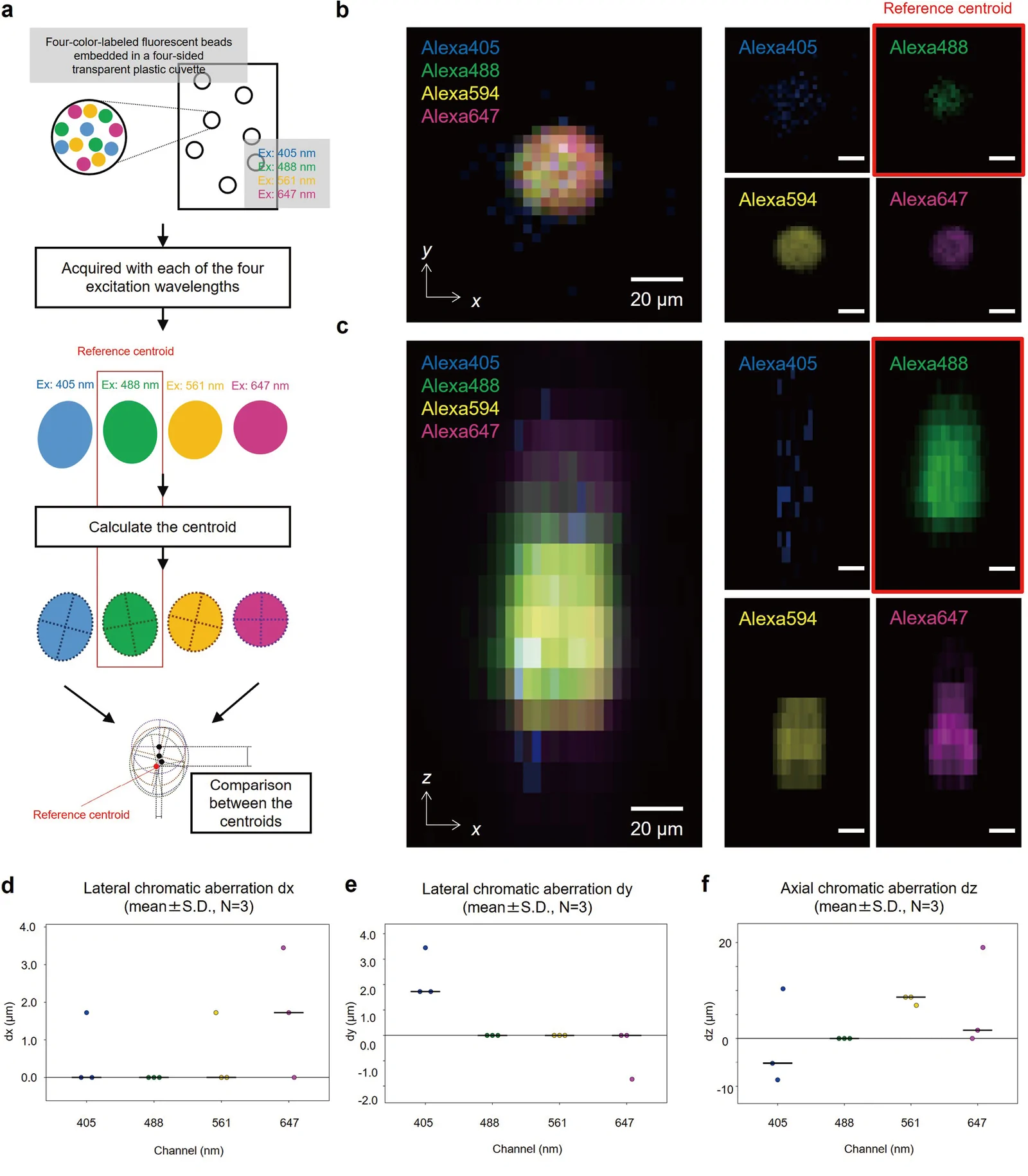
Chromatic aberration evaluation of the extended descSPIM system using a multicolor optical phantom **a.** Schematic of the workflow for chromatic aberration evaluation using a custom multicolor optical phantom. Protein A/G agarose beads were labeled with four fluorophore-conjugated secondary antibodies, imaged at each excitation wavelength, manually outlined, and analyzed by centroid measurement to determine chromatic displacements relative to the 488 nm reference channel. **b.** Evaluation of lateral (*xy*) chromatic aberration using a multicolor optical phantom, shown as a four-color merged image and the corresponding single-channel lateral images for each excitation wavelength. **c.** Evaluation of axial (*xz*) chromatic aberration using the same multicolor optical phantom, shown as a four-color merged image and the corresponding single-channel axial images for each excitation wavelength. **d.** Quantitative evaluation of lateral chromatic aberration in the xy plane, expressed as centroid displacement Δ*x* relative to the 488 nm channel. **e.** Quantitative evaluation of lateral chromatic aberration in the *xy* plane, expressed as centroid displacement Δ*y* relative to the 488 nm channel. **f.** Quantitative evaluation of axial chromatic aberration along the z axis, expressed as centroid displacement Δ*z* relative to the 488 nm channel. Data were obtained from three independent beads (N = 3). The bar indicates the median, and individual data points represent measured values from each bead. The horizontal line indicates zero displacement relative to the reference channel. The larger dispersion observed for Δ*z*, compared with Δ*x* and Δ*y*, is likely attributable to the elongated axial point spread function, which reduces centroid precision along the *z* axis. Nevertheless, the measured centroid displacements were within the measured lateral and axial resolution of the system (8.56 µm laterally and 25.92 µm axially), indicating minimal chromatic aberration across excitation wavelengths from 405 to 647 nm.

Following the addition of the 405 nm channel, the broader wavelength range necessitated an evaluation of potential chromatic aberration. Therefore, we subsequently assessed the chromatic aberration of this extended system. To this aim, we established an aberration measurement method using a custom multicolor optical phantom consisting of Protein A/G agarose beads labeled with AF405-, 488-, 594-, and 647-conjugated secondary antibodies (Figure 2a). The *xy* and *xz* images of a single bead demonstrated nearly complete overlap of fluorescent signals from all four channels, suggesting minimal chromatic aberration (Figure 2b, c). To further quantify the degree of lateral and axial chromatic aberration, we measured the centroid displacements of individual beads (Δ*x*, Δ*y*, and Δ*z*) across excitation wavelengths from 405 nm to 647 nm. These displacements were plotted relative to the 488 nm channel as a reference (Figure 2d-f). Individual measured displacements were plotted as data points, with bars indicating the median values. Importantly, the measured displacements were smaller than the corresponding measured resolution values of the system (8.56 µm laterally and 25.92 µm axially), indicating that chromatic aberration in this proposed system is largely negligible for cellular-resolution multicolor imaging within this wavelength range.

### Four-color whole-embryo imaging of an E12.5 mouse embryo using the system

**Figure 3:**
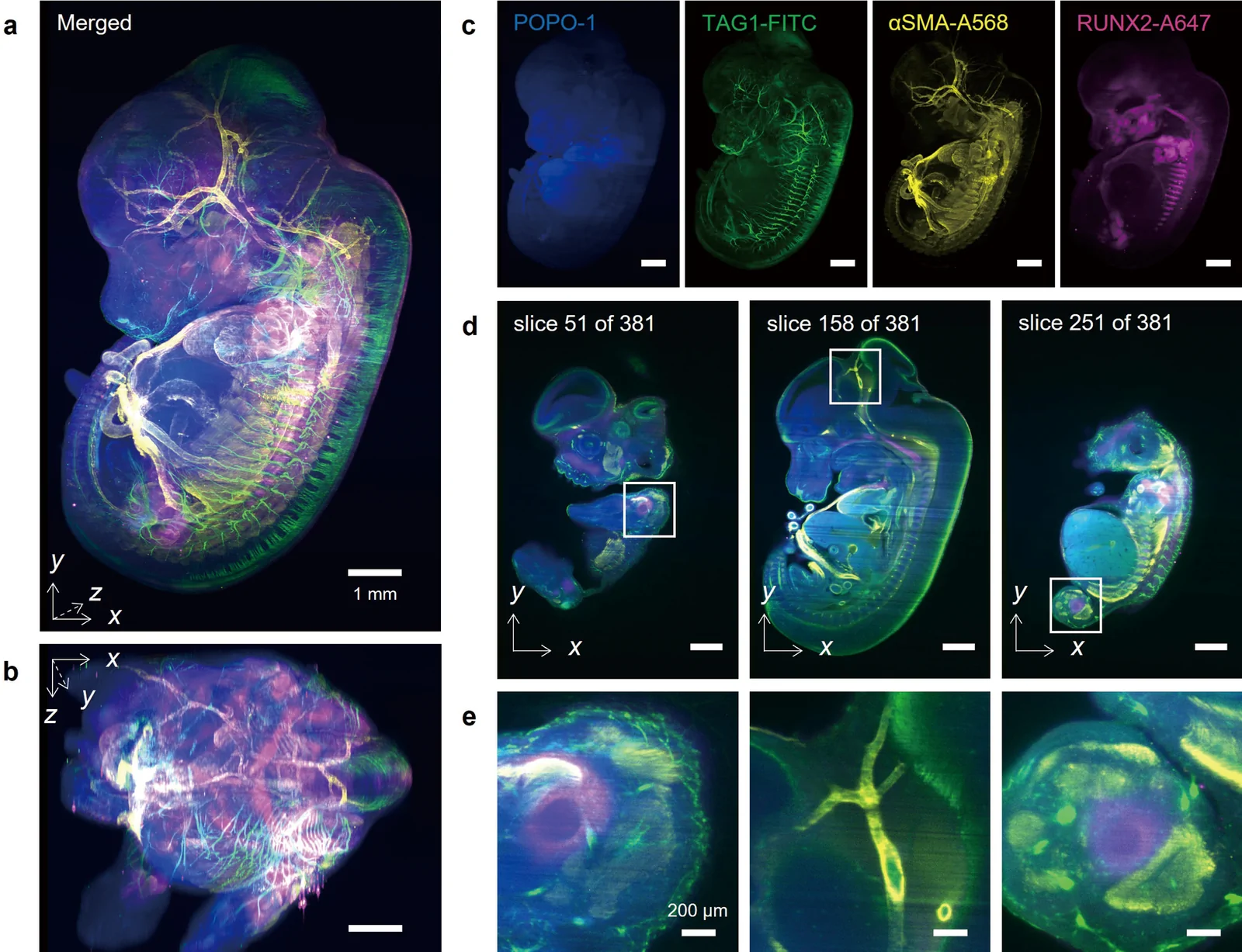
Four-color whole-embryo imaging of an E12.5 mouse embryo using the descSPIM **a.** Maximum intensity projection (MIP) of a whole E12.5 mouse embryo acquired using the descSPIM. Nuclei were labeled with POPO-1 (blue), and immunostaining was performed for TAG1-FITC (green), αSMA-AF568 (yellow), and RUNX2-AF647 (magenta). **b.** Orthogonal MIP views derived from the same *z*-stack. **c.** Single-channel MIPs for each fluorescence channel. **d.** Representative single optical sections prior to MIP at slices 51, 151, and 258 out of a total of 381 *z*-slices. **e.** Magnified views of selected regions of interest (ROIs) corresponding to the slices shown in d. Scale bars, 1 mm (a-d) and 200 µm (e).

To demonstrate the applicability of the extended descSPIM to intact biological specimens, we performed four-color whole-body imaging of cleared E12.5 mouse embryo (~10 × 5 × 5 mm, crown-rump length × width × depth) (Figure 3). Nuclear staining with POPO-1 provided a global anatomical reference by visualizing the spatial distribution of cell nuclei throughout the embryo (Figure 3a). The brights signal from the UV-excited fluorophores used in our cleared mouse embryo enabled the detection of all cell distributions with nearly uniform intensity from the surface to the deep regions. This is attributable to both the properties of the cleared specimen and the enhanced performance of CUBIC- HistoVIsion2.0. Immunostaining with an anti-TAG1- FITC antibody revealed neuronal structures and axonal processes, showing the organization and extension of neural networks. Simultaneously, an anti-αSMA-AF568 antibody identified smooth muscle and vascular structures, while an anti-RUNX2-AF647 antibody labeled cells of the osteogenic lineage. (Figure 3a, c). The orthogonal views of the dataset demonstrated minimal misalignment across the fluorescence channels along the axial axis, confirming consistent multicolor registration throughout the imaging depth (Figure 3b). Representative single optical sections acquired at different depths within the embryo further showed that multicolor signals remained well aligned (Figure 3d). In addition, magnified views of selected regions of interest (ROIs) revealed the local spatial organization of neuronal processes, smooth muscle structures, and osteogenic cells within the same tissue microenvironment (Figure 3e). Together, these results demonstrate that the extended descSPIM enables robust four-color whole-embryo imaging with minimal inter-channel misalignment, allowing integrated visualization of multiple developmental features within a single intact embryo (Figure 3).

## Discussion

In this study, we presented an extended descSPIM model and demonstrated that its optical configuration maintains mostly negligible chromatic aberration across a broad range of excitation wavelengths. Based on this optical validation, we performed simultaneous four-color light-sheet imaging of a whole cleared E12.5 mouse embryo. These results indicate that the descSPIM platform supports practical multicolor imaging without the need for complex optical corrections, while keeping its compact and accessible design. This capability is expected to support a wide range of research applications such as developmental biology and cancer biology requiring the integrated 3D visualization of complex tissues. Additionally, the optical phantom-based workflow developed here for measuring chromatic aberration (Figure 2) may also be extended to other systems. However, in the case of higher-magnification microscopes, 3D images of sufficiently smaller beads should be used for precise evaluations (for example, submicrometer beads). In the present analysis, fluorescent spots in the optical phantom that could be detected across all four excitation wavelengths were selected, although the fluorescence signal under 405-nm excitation was relatively weak. Under brighter imaging conditions, intensity-weighted centroid estimation using Fiji functions or plug-ins may further improve object-center determination^10)^. It should be noted that axial chromatic shifts may also be influenced by wavelength-dependent dispersion of the imaging medium^14)^.

Looking forward, we anticipate a further increase in the number of simultaneously observable molecular targets. Additional developments in both instrumentation and labeling, such as multispectral acquisition^15, 16)^, refined optical control, and the use of long-Stokes-shift dyes, will be essential for achieving highly multiplexed imaging. Successfully integrating these more complex techniques into the accessible descSPIM framework remains a key focus for future development.

## Data availability

The datasets generated and/or analyzed during the current study are available from the corresponding author upon reasonable request.

## Author contributions

EAS, KO, and YN designed the project. YN and YS prepared the samples. YN performed experimental investigations, including optimization of imaging conditions, microscope construction, and image acquisition. YN drafted the manuscript. EAS, KO, SJE, and HB reviewed and edited the manuscript.

## Conflicts of interest statement

EAS has received long-term large-scale research funding (FY2024-2028) from the Nakatani Foundation and concurrently serves as an executive officer of CUBICStars Inc. EAS is also a co-inventor on patents and patent applications owned by RIKEN covering the CUBIC reagents and is employed by CUBICStars Inc., which offers services based on CUBIC technology. The remaining authors declare no competing interests.

## References

[1] Voie AH, Burns DH, Spelman FA: Orthogonal-plane fluorescence optical sectioning: Three-dimensional imaging of macroscopic biological specimens. J Microsc, 1993; 170: 229-236. 8371260 10.1111/j.1365-2818.1993.tb03346.x

[2] Dodt HU, Leischner U, Schierloh A, et al: Ultramicroscopy: three-dimensional visualization of neuronal networks in the whole mouse brain. Nat Methods, 2007; 4: 331-336. 17384643 10.1038/nmeth1036

[3] Huisken J, Swoger J, Del Bene F, Wittbrodt J, Stelzer EHK: Optical Sectioning Deep Inside Live Embryos by Selective Plane Illumination Microscopy. Science, 2004; 305: 1007-1009. 15310904 10.1126/science.1100035

[4] Keller PJ, Schmidt AD, Wittbrodt J, Stelzer EHK: Reconstruction of Zebrafish Early Embryonic Development by Scanned Light Sheet Microscopy. Science, 2008; 322: 1065-1069. 18845710 10.1126/science.1162493

[5] Pitrone PG, Schindelin J, Stuyvenberg L, et al: OpenSPIM: an open-access light-sheet microscopy platform. Nat Methods, 2013; 10: 598-599. 23749304 10.1038/nmeth.2507 PMC7450513

[6] Gualda EJ, Vale T, Almada P, Feijó JA, Martins GG, Moreno N: OpenSpinMicroscopy: an open-source integrated microscopy platform. Nat Methods, 2013; 10: 599-600. 23749300 10.1038/nmeth.2508

[7] Voigt FF, Kirschenbaum D, Platonova E, et al: The mesoSPIM initiative: open-source light-sheet microscopes for imaging cleared tissue. Nat Methods, 2019; 16: 1105-1108. 31527839 10.1038/s41592-019-0554-0 PMC6824906

[8] Vladimirov N, Voigt FF, Naert T, et al: Benchtop mesoSPIM: a next-generation open-source light-sheet microscope for cleared samples. Nat Commun, 2024; 15: 2679. 38538644 10.1038/s41467-024-46770-2 PMC10973490

[9] Otomo K, Omura T, Nozawa Y, et al: descSPIM: an affordable and easy-to-build light-sheet microscope optimized for tissue clearing techniques. Nat Commun, 2024; 15: 4941. 38866781 10.1038/s41467-024-49131-1 PMC11169475

[10] Schindelin J, Arganda-Carreras I, Frise E, et al: Fiji: an open-source platform for biological-image analysis. Nat Methods, 2012; 9: 676-682. 22743772 10.1038/nmeth.2019 PMC3855844

[11] Susaki EA, Shimizu C, Kuno A, et al: Versatile whole-organ/body staining and imaging based on electrolyte-gel properties of biological tissues. Nat Commun, 2020; 11: 1982. 32341345 10.1038/s41467-020-15906-5 PMC7184626

[12] Matsumoto K, Mitani TT, Horiguchi SA, et al: Advanced CUBIC tissue clearing for whole-organ cell profiling. Nat Protoc, 2019; 14: 3506-3537. 31748753 10.1038/s41596-019-0240-9

[13] Susaki EA, Kurusu R, Saito Y: CUBIC-HistoVIsion2.0, published on Feb 25, 2026. Published online February 25, 2026. Accessed March 7, 2026. https://www.protocols.io/view/cubic-histovision2-0-n92ld1wyxl5b/v1

[14] Schadwinkel H, Selchow O, Weisshart K, Haarstrich J, Birkenbeil J: ZEISS Lightsheet 7: How to Get Best Images with Various Types of Immersion Media and Clearing Agents. Carl Zeiss Microscopy GmbH, 2020.

[15] Jahr W, Schmid B, Schmied C, Fahrbach FO, Huisken J: Hyperspectral light sheet microscopy. Nat Commun, 2015; 6: 1-7. 10.1038/ncomms8990 PMC4569691 26329685

[16] Wang P, Kitano M, Keomanee-Dizon K, Truong TV, Fraser SE, Cutrale F: A single-shot hyperspectral phasor camera for fast, multi-color fluorescence microscopy. Cell Rep Methods, 2023; 3. Accessed December 16, 2025. https://www.cell.com/cell-reports-methods/fulltext/S2667-2375(23)00056-5 10.1016/j.crmeth.2023.100441 PMC10162951 37159674

